# The sino-nasal warzone: transcriptomic and genomic studies on sino-nasal aspergillosis in dogs

**DOI:** 10.1038/s41522-020-00163-7

**Published:** 2020-11-12

**Authors:** I. D. Valdes, A. B. P. Hart de Ruijter, C. J. Torres, J. C. A. Breuker, H. A. B. Wösten, H. de Cock

**Affiliations:** 1 Microbiology, Department of Biology, Utrecht University, Utrecht, The Netherlands; 2 Institute of Biomembranes, Utrecht University, Utrecht, The Netherlands

**Keywords:** Biofilms, Pathogens, Molecular evolution, Microbial genetics

## Abstract

We previously showed that each dog with chronic non-invasive sino-nasal aspergillosis (SNA) was infected with a single genotype of *Aspergillus fumigatus*. Here, we studied the transcriptome of this fungal pathogen and the canine host within the biofilm resulting from the infection. We describe here transcriptomes resulting from natural infections in animal species with *A. fumigatus*. The host transcriptome showed high expression of IL-8 and alarmins, uncontrolled inflammatory reaction and dysregulation of the Th17 response. The fungal transcriptome showed in particular expression of genes involved in secondary metabolites and nutrient acquisition. Single-nucleotide polymorphism analysis of fungal isolates from the biofilms showed large genetic variability and changes related with adaptation to host environmental factors. This was accompanied with large phenotypic variability in in vitro stress assays, even between isolates from the same canine patient. Our analysis provides insights in genetic and phenotypic variability of *Aspergillus fumigatus* in biofilms of naturally infected dogs reflecting in-host adaptation. Absence of a Th17 response and dampening of the Th1 response contributes to the formation of a chronic sino-nasal warzone.

## Introduction

Infections with *Aspergillus fumigatus* occur in a wide variety of animal species including humans, birds, cows, horses, cats, and dogs^1,2^. In dogs, sino-nasal aspergillosis (SNA) is the most common *Aspergillus* infection^3^. The fungus can reside in the host for an extended period of time (e.g., months) before manifesting clinical signs, which include sneezing, mucoid nasal discharge, nasal depigmentation, epistaxis, and turbinate destruction^2,4^. This relative long build-up time facilitates the fungus to form a fungal biofilm covering the sino-nasal area up to the frontal sinus before the disease is diagnosed^2^. Proper diagnosis of SNA requires a combination of computed tomography (CT), rhinoscopy, histopathology, cytology, fungal culturing, and serology^2^. Topical administration of azole drugs remains the most widely used and successful treatment for SNA in dogs although reports of its use are limited^2,3,5^. SNA has been associated with upregulation of Th1 cytokines like interleukin (IL)-8 and TNF-α^6–8^. This may indicate a localized response to the fungus to restrain the dispersal and development of an invasive infection. Yet, a high level of inflammation and incapability to clear the infection has been suggested to be due to the upregulation of anti-inflammatory molecules such as transforming growth factor-β and IL-10^7,8^.

To date, only in vivo *A. fumigatus* transcriptomes are available from murine infection models^9,10^ but these may not be representative of natural infections. Here, we describe for the first-time transcriptomic profiling of natural *A. fumigatus* infections in the sinus of canine hosts using RNA sequencing. In all cases we studied the fungus that had formed a mature biofilm. We previously showed fungal heterogeneity at the level of pigmentation between isolates from a single dog as compared with isolates from human as well as indoor and outdoor substrates^4^. This suggested that the dog sinus is a highly selective environment inducing in-host adaptations resulting in phenotypic evolution. The fungus is expected to experience among others nutrient stress, host immune responses, and limited oxygen availability as selection pressures. The results described in this paper show that the sino-nasal mucosal surface is a warzone between *A. fumigatus* and the host tissue. The host fights the fungal biofilm using nutritional immunity, and inflammatory and Th1 responses, and on the other battle side, the adaptive responses of *A. fumigatus* enable growth of the pathogen and contribute to the suppression of the host immune response.

## Results

### Dog transcriptome

**Fig. 1:**
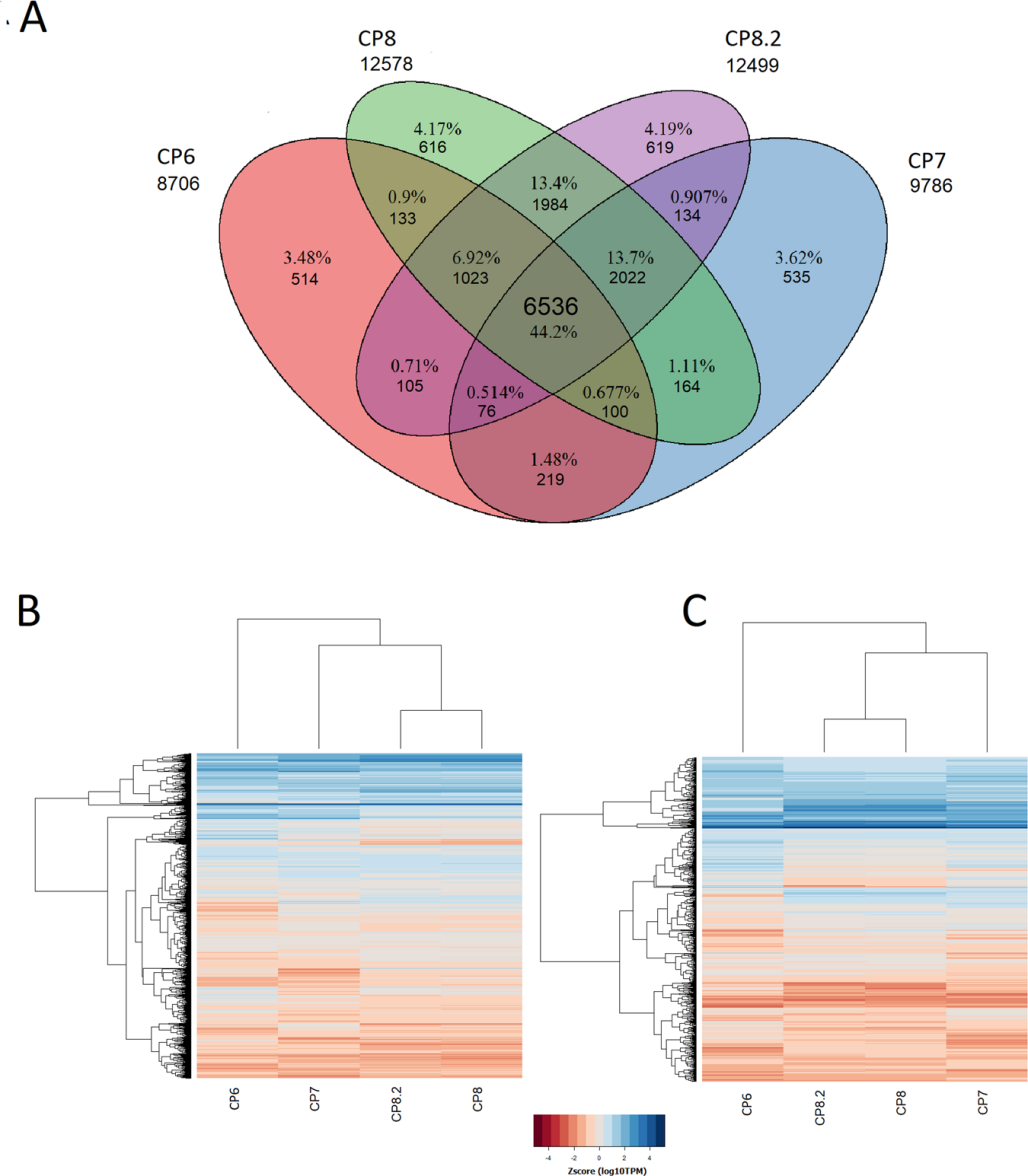
Transcriptomic profiles. Transcriptome profiles of four fungal plaques of three different dogs with SNA. Venn diagram **a** showing number and percentage of shared transcripts (TPM > 1) between all four fungal plaques and heatmaps with hierarchical clustering of the 6536 shared transcripts **b** and the 660 transcripts mapped to the InnateDB database^11^ **c**. Number of transcripts with TPM > 1 is shown under each patient code in **a**.

**Fig. 2:**
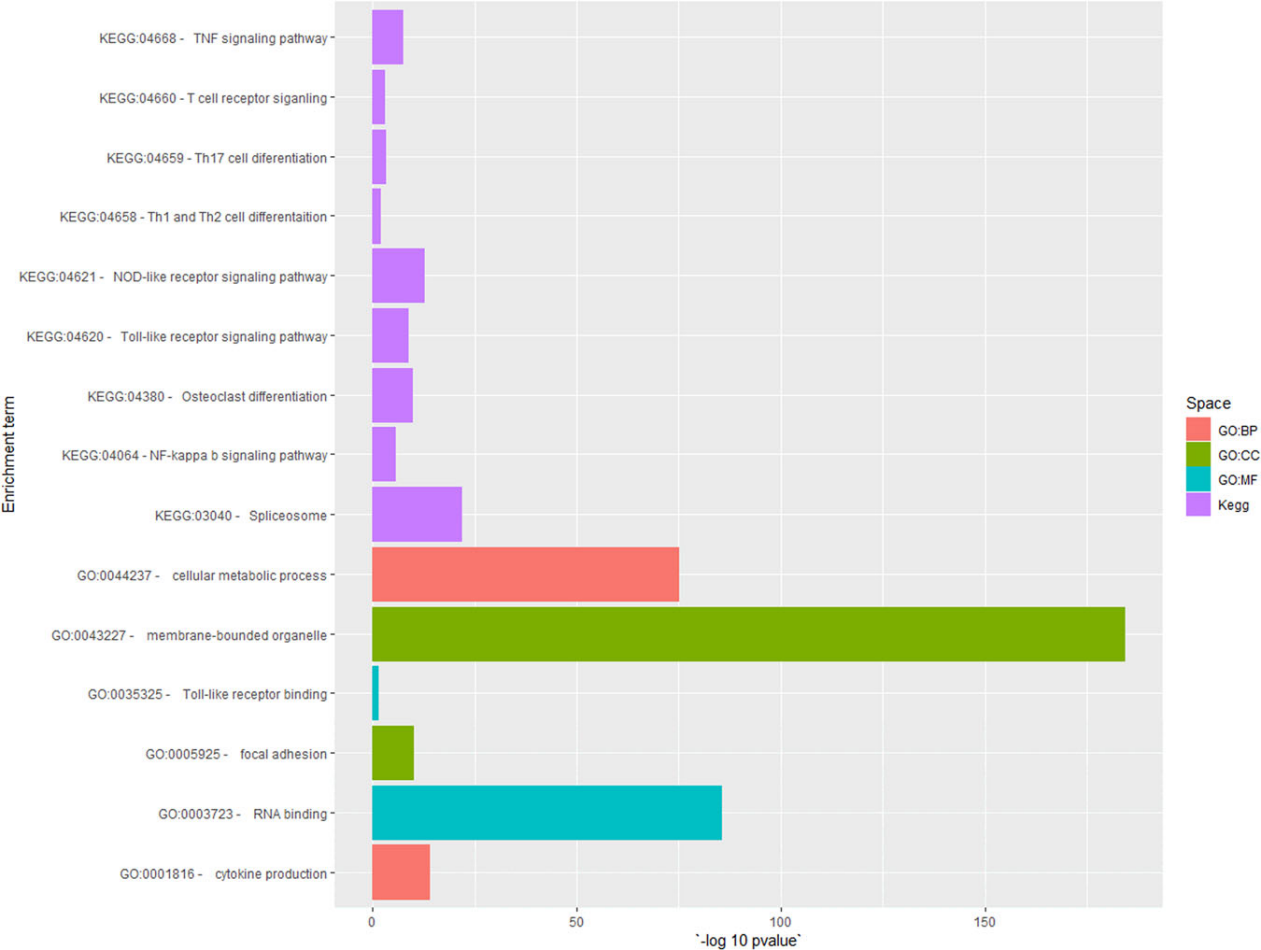
GO and KEGG term enrichment analysis. GO and KEGG term enrichment analysis of the 6536 shared expressed transcripts in four fungal plaques from three dogs with SNA. BP indicates biological process, CC cellular component and MF molecular function (for full list see Supplementary data set 1).

Read mapping of the four fungal plaques from three different dogs with SNA to the dog reference transcriptome (38019 transcripts) resulted in 8706–12578 transcripts with TPM > 1 per sample (Fig. 1A). About 44% (i.e., 6536) of the transcripts were shared between the four plaques (Fig. 1A). This number corresponds to 17% of the total transcriptome of *C. lupus familiaris*. The relatively low number of expressed transcripts reflects the low amount of canine RNA present in the fungal plaques (i.e., 1–17% of a total of 3–6 × 10^7^ reads) (Supplementary data set 1). The 6536 shared transcripts were considered as the SNA dog transcriptome and were used for further analysis. Hierarchical clustering of the shared transcriptomes showed that the two samples derived from the same dog, CP8 and CP8.2, clustered together (Fig. 1B). This suggests that the four transcriptomes share many features even though the fungal pathogens have different genotypes^4^. GO and KEGG term analysis (Fig. 2 and Supplementary data set 1; see Goprofiler) showed significant enrichment in the shared expressed transcripts in general terms like cellular metabolic process (GO:0044237), and RNA binding (GO:0003723). In addition, immune response terms like Toll-like receptor binding (GO:0035325), cytokine production (GO:0001816), Th1 and Th2 cell differentiation (KEGG:04658), as well as osteoclast differentiation (KEGG: 03480) were overrepresented. The latter is related to bone destruction and remodeling.

Ranking of the transcripts by their mean TPM revealed that the top 50 had 20 transcripts related to immune response with IL-8 as the highest ranked transcript (Supplementary data set 1). Also, high amounts of transcripts related to nutritional immunity (e.g., ferritin, and S100A8, A9, and A12) were detected.

Mapping the SNA dog transcriptome to the immune database InnateDB^11^ resulted in 660 hits (i.e., 11% of the 6536 shared transcripts) (Supplementary data set 1). As observed with the general SNA transcriptome (Fig. 1B), the InnateDB transcripts derived from the same dog (CP8, CP8.2) clustered (Fig. 1C). This included among others 6 interleukins (IL-1, 8, 10, 15, 16, 18, 23A), 12 interleukin receptors (IL 1R2, 2RG, 4R, 10RA, 13RA1, 15RA, 17RA/B, 18R1, 22RA2, 27RA, 31RA), seven toll-like receptors (TLR-1, 2, 4, 6, 7, 8, 10), and other important genes such as nucleotide-binding oligomerization domain containing protein 2, and Interferon gamma (Supplementary data set 1). The Bruvo genetic distance between isolates from these patients differed 0.46 as determined by STR*Af*^4^. This indicates that the host and not the genotype of *A. fumigatus* has strongest impact on the immune response.

**Fig. 3:**
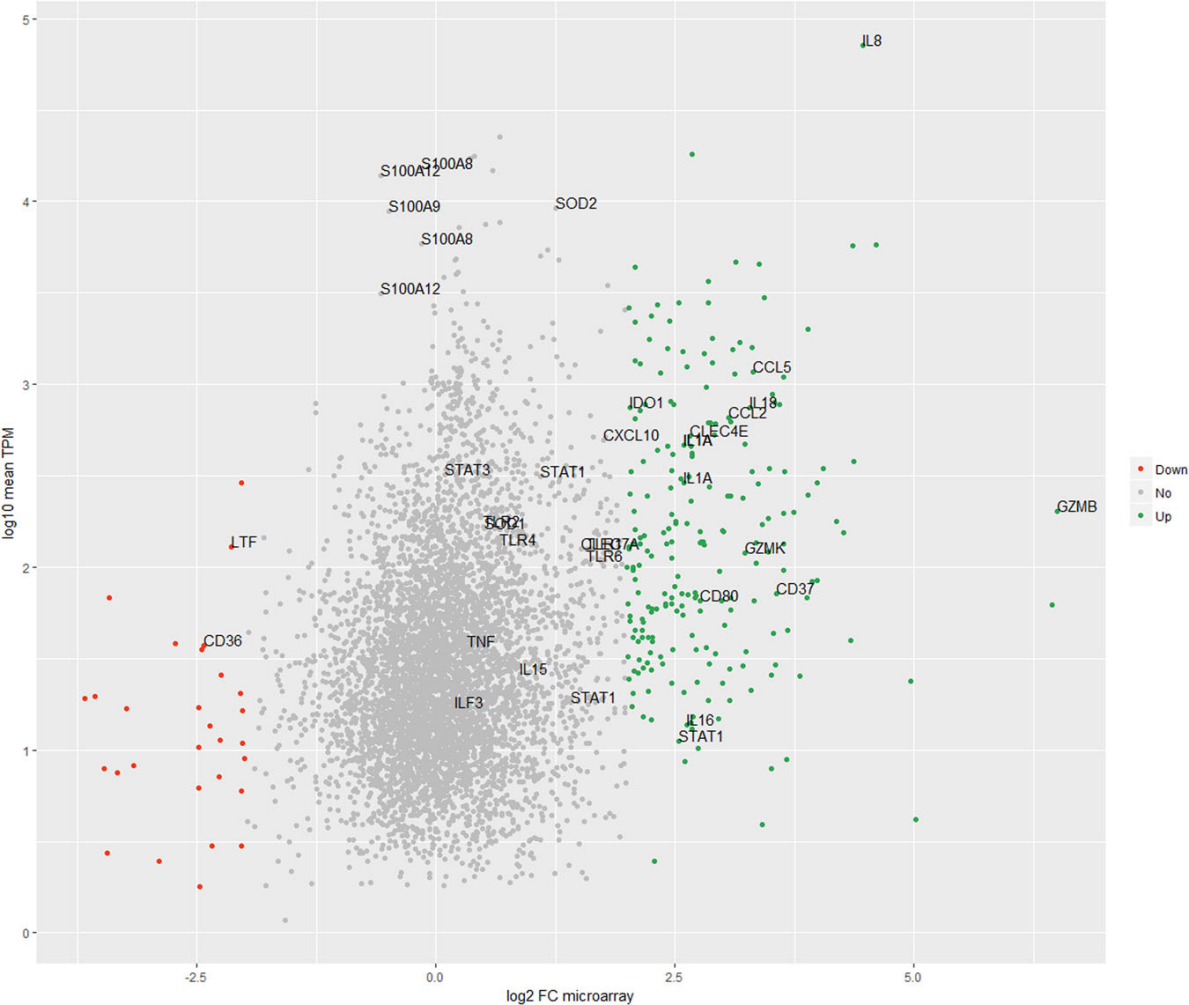
Comparison of RNAseq data. Comparison between the RNAseq data of this study and the microarray from^6^. Shared transcripts between our RNAseq data and found to be upregulated, downregulated, and not differentially expressed (log2 FC ≥ 2) in the microarray are in green, red, and gray, respectively. Transcripts mentioned in the Results and/or Discussion section are labeled.

We compared our RNAseq results with previously published canine microarray data describing differential gene expression of healthy dogs versus dogs with SNA^6^. We used a log2 fold change ±2 observed in the microarray data for the identification of differentially expressed genes. This resulted in 948 out of 5551 differentially expressed transcripts. Of these differentially expressed transcripts, 307 transcripts were also expressed in our RNAseq data (Supplementary data set 1). Enrichment analysis of these 307 differentially expressed genes showed that upregulated genes (264) were enriched in immune-related terms like superoxide anion generation (GO:0042554), phagocytosis (GO:0006909), and immune system process (GO:002376). (Supplementary data set 1). Interestingly, 15 transcripts within the top 50 highest expressed genes detected by RNAseq analysis were differently expressed in the microarray (Supplementary data set 1). Genes like S100 A12, A9, A8, and SOD2 were not differentially expressed in the microarray. In contrast, IL-8 and IDO1 were upregulated and LTF was downregulated (Fig. 3).

### Fungal transcriptome

A total of 9213 genes, representing 92.8% of the genes of the published Af293 genome^12^ were found to be expressed in the SNA samples (TPM > 1) (Supplementary data set 2). Of these genes, 8029 (~87%) were shared among all four fungal plaques. We observed a high variation of expression levels between these samples, i.e., in some cases >1000-fold. Therefore, the coefficient of variation (CV) of the log10 TPM (CV = standard deviation in four samples/mean in four samples) of the 8029 expressed genes was calculated and classified as highly stable expressed (CV < 0.05), medium stable expressed (0.5 > CV > 0.05) and highly variable expressed (CV > 0.5). These groups compromised 13, 81, and 6% of the genes, respectively.

**Fig. 4:**
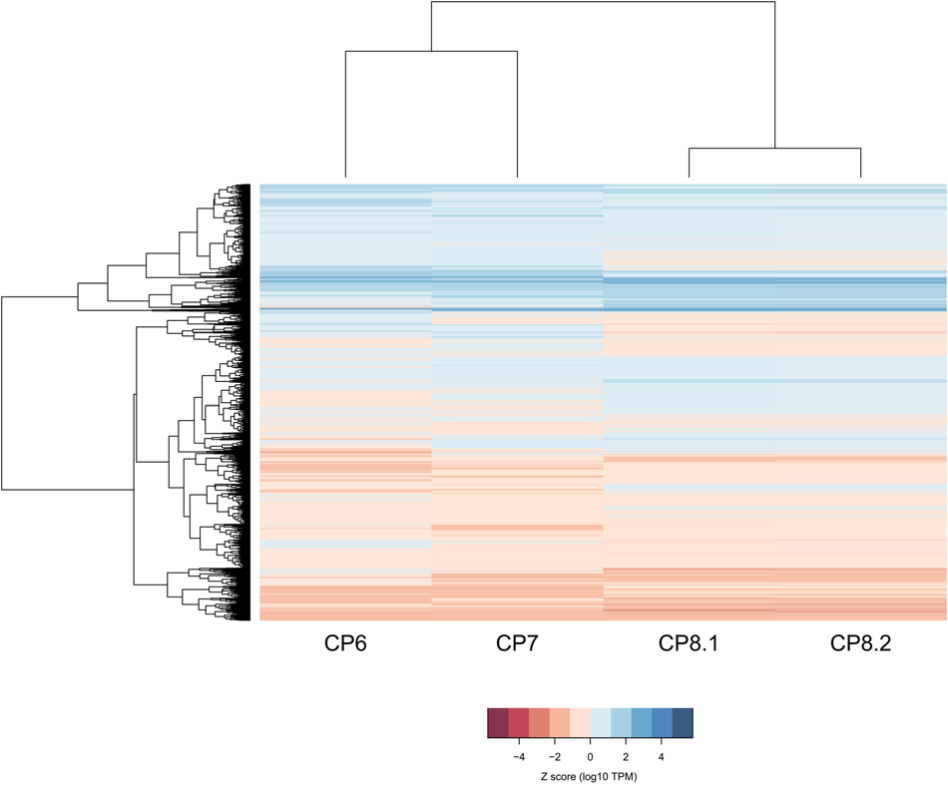
Heatmap of expressed and shared fungal genes. Heatmap clustering expression of shared genes expressed in the fungal plaques of CP6, CP7, CP8, and CP8.2.

Hierarchical clustering of the transcriptomes of shared genes in each of the fungal plaques of the three dogs revealed that fungal plaques from the same dog (CP8 and CP8.2) have a more similar expression profile than those of CP6 and CP7 (Fig. 4). Notably, the expression profiles of CP6 and CP8 are most different although the fungal isolates derived from these two plaques have the same genotype^4^. A similar distribution of gene expression was observed when shared expressed genes were compared that are related to secondary metabolism^13^ (Supplementary Fig. 1A), stress-response genes^14^ (Supplementary Fig. 1B), pathogen–host interaction^15^ (Supplementary Fig. 1C) and transcription factors (TFs) related to virulence^16^ and pathogenesis combined with reproduction and stress response^17^ (Supplementary Fig. 1D). Again, the transcriptomes of CP6 and CP8 were most distinct although these strains have the same genotype. Apparently, the host is more directive in gene expression.

**Fig. 5:**
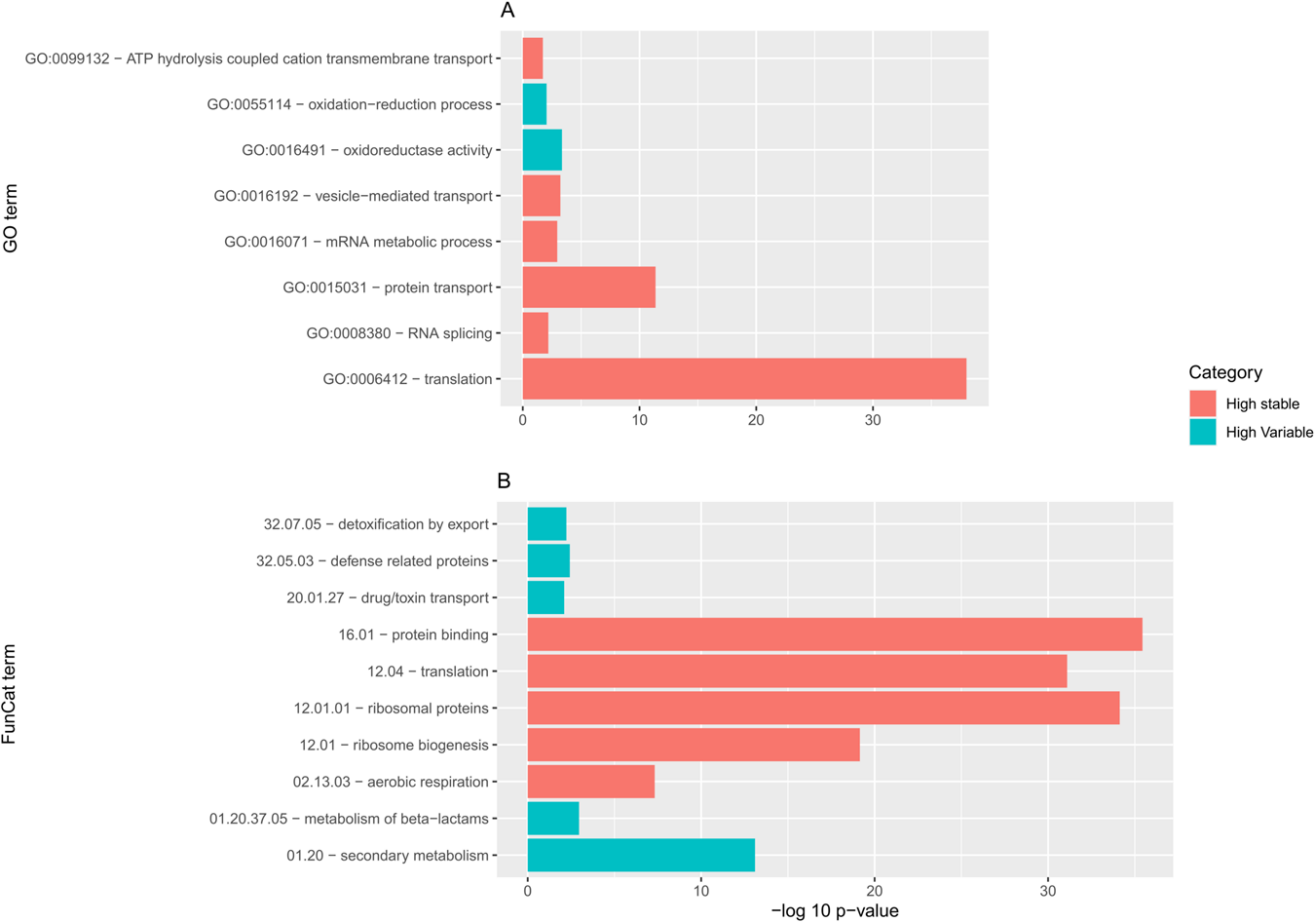
GO and FunCat analysis. GO **a** and FunCat (**b**; representing the first five terms) enrichment of shared expressed genes with low and high variation in expression. See Supplementary data set 2 for the complete enrichment analysis.

Fifteen GO terms were enriched within the 1046 expressed genes with low variability, most of them belonging to general categories like mRNA metabolic process (GO:0016071) and translation (GO:0006412) (Supplementary data set 1). In agreement, FunCat ontology analysis also showed enrichment in categories like translation (12.04), and ribosomal proteins (12.01.01) (Fig. 5). Enrichment of genes categorized as having a medium variability in expression showed general categories like cellular protein catabolic process (GO:0044257), cellular process (GO:0009987) and binding (GO:0005488). On the other hand, the 514 genes with highly variable expression were only enriched in the GO terms oxidation-reduction process (GO:0055114) and oxidoreductase activity (GO:0016491) (Supplementary data set 2). In the case of FunCat analysis, this group of genes showed enrichment in secondary metabolism (01.20), defense related proteins (32.05.03), resistance proteins (32.05.01) and other terms related to virulence (Fig. 5B; Supplementary data set 2).

### Whole-genome sequencing of fungal isolates

**Fig. 6:**
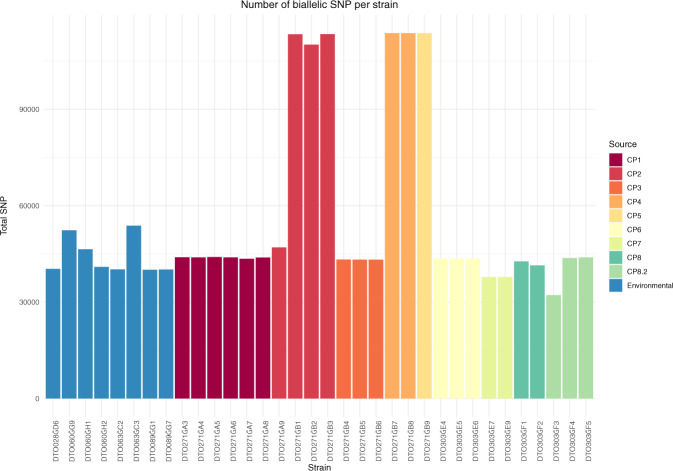
SNP analysis. Number of biallelic SNPs per *A. fumigatus* isolate.

Genomic DNA of 34 isolates was sequenced with an average 23.61-fold coverage (Supplementary data set 3) and compared with the Af293 reference genome^12^. The variation of the genomes of the isolates ranged between 0.11% and 0.3% relative to Af293. The SNPs were regularly distributed along the 29,388,377 bp Af293 genome. Biallelic SNPs were the most prevalent variant (average number 56064), whereas multiallelic SNPs (average number 219) were much less frequently detected (Supplementary data set 3). Interestingly, the genomes of six isolates from a total of three dogs contained 2.5-fold more biallelic SNPs when compared to the other dog and environmental isolates (Fig. 6). This high-SNP number was generally distributed along the genome (Supplementary data set 3). Three out of the six isolates had been isolated from dog CP2, from which also one isolate had been obtained with a low SNP incidence (Fig. 6).

### Phylogeny

**Fig. 7:**
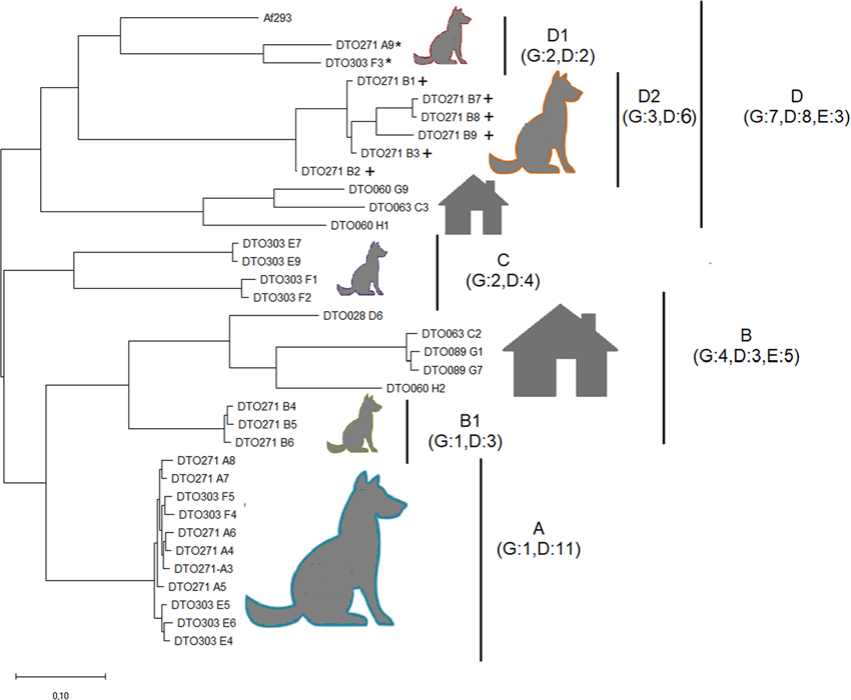
SNP-based phylogenetic tree. SNP-based phylogenetic tree of the 26 isolates from eight SNA patients and from eight environmental isolates. The tree was inferred by using the maximum likelihood method based on the general time-reversible model. The tree with the highest log likelihood (−49,077.29) is shown and drawn to scale, with branch lengths measured in the number of substitutions per site. There was a total of 3826 nucleotide positions in the final data set. Evolutionary analyses were conducted in MEGA X^71^. Selected clades (A, B, C, D) and sub clades (B1, D1, D2) are depicted with letters and numbers. The dog and environmental isolates are indicated with a dog and house pictogram, respectively, whereas G, D, and E indicate the number of STR*Af* genotypes, dog isolates, and environmental isolates, respectively. Two isolates that were not grouped with isolates from the same STR*Af* genotype are denoted by a star (*). Isolates with a notable high-SNP density are denoted by a plus (+) symbol.

Clades A–D were identified using a SNP-based phylogeny (Fig. 7). Clades B and D contain both environmental and dog isolates, whereas clades A and C contain canine isolates only. The isolates of clade A are derived from patients CP1, CP6, and CP8.2 and have a very small genetic distance based on the SNP-based phylogenetic tree. This is in agreement with genotyping based on STR*Af*^4^ (Supplementary data set 3). Clade B contained isolates of CP3 only, whereas clade C contained isolates of CP7 and CP8.2 and clade D of CP2, CP4, CP5, and CP8. In general, clustering was in line with microsatellite analysis with two exceptions. Isolates DTO 271-A9 (from CP2) and DTO 303-F3 (from CP8.2) are now in subclade D1 but were expected to be in subclade D2 and cluster A, respectively, based on the microsatellite analysis^4^.

### SNPs in secondary metabolite clusters

**Fig. 8:**
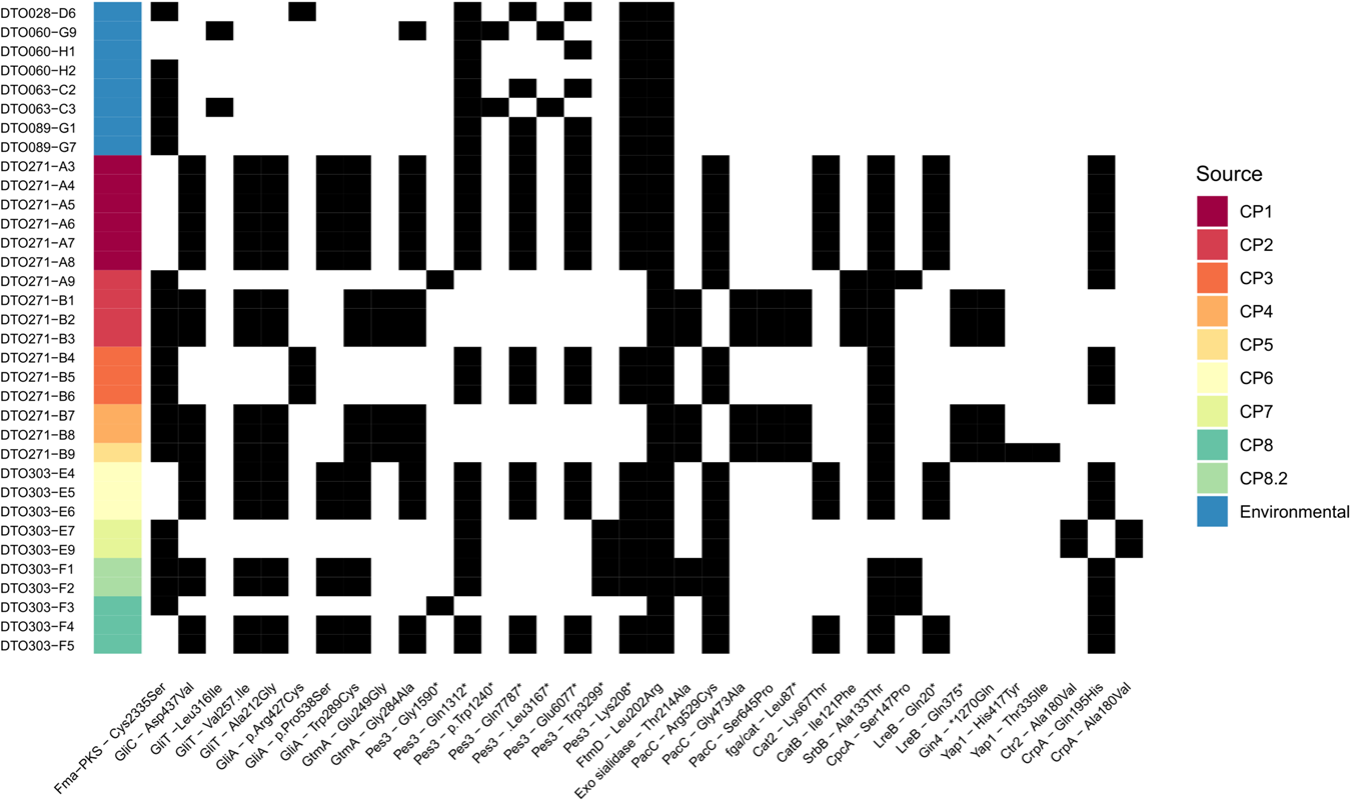
View of SNPs per strain. SNPs present in an isolate are indicated by black shading of the cell. The origin of the isolates is depicted as a colored bar to the left.

Non-core SNPs of dog isolates with high impact are enriched in secondary metabolic gene clusters. A missense mutation (Cys2335Ser) in Fma-PKS (Afu8g00370) (Table 1) encoding a polyketide synthase (PKS) of the fumagillin cluster (cluster 30) is present in 15 out of the 26 isolates present in clades B, C, and D but absent in clade A (CP1, CP6, and CP8.2). This nsSNP (non-synonymous SNP) is not present in two out of eight environmental isolates (i.e., DTO 060-G9 and DTO 063-C3) (Fig. 8). Fumagillin is one of the mycotoxins of *A. fumigatus* and whether this SNP affects the fumagillin function needs to be addressed.

**Table 1:** Selected SNPs with resulting amino-acid change, their potential effect and frequency in environmental and dog isolates.

Process/molecule	Gene	Nucleotide	Protein	Environmental	Dog	Possible effect
Fumagillin	Fma-PKS (Afu8g00370)	c.7003 T > A	p.Cys2335Ser	6	15	Inhibition of production of metabolite
Gliotoxin	GliC (Afu6g09670)	c.1310 A > T	p.Asp437Val	0	19	Production of intermediate species changed
	GliT(Afu6g09740)	c.946 C > A	p.Leu316Ile	1	19	
		c.769 G > A	p.Val257.Ile	0	19	Production of active gliotoxin changed
		c.635 C > G	p.Ala212Gly	0	19	
	GliA (Afu6g09710)	c.867 G > T	p.Trp289Cys	1	13	Transport of gliotoxin changed
		c.1279 C > T	p.Arg427Cys	1	3	
		c.1612c > T	p.Pro538Ser	0	19	
	GtmA (Afu2g11120)	c.746 A > G	p.Glu249Gly	0	6	Detoxification of gliotoxin changed
		c.851 G > C	p.Gly284Ala	1	17	
NRP	Pes3 (Afu5g12730)	c.4768 G > T	p.Gly1590*	0	2	Inhibition of production of metabolite
		c.3934 C > T	p.Gln1312*	8	18	
		c.3719 G > A	p.Trp1240*	2	0	
		c.23359 C > T	p.Gln7787*	4	14	
		c.9500 T > A	p.Leu3167*	2	0	
		c.18229 G > T	p.Glu6077*	5	14	
		c.9896 G > A	p.Trp3299*	0	4	
		c.9896 G > A	p.Lys208*	8	18	
Fumitremorgin	FtmD (Afu8g00200)	c.605 T > G	p.Leu202Arg	8	26	Production of metabolite
Exo sialidase	Afu4g13800	c.640 A > G	p.Thr214Ala	0	7	Host recognition changed
pH response	PacC (Afu3g11970)	c.1585 C > T	p.Arg529Cys	0	20	Changes in response to pH
		c.1418 G > C	p.Gly473Ala	0	6	
		c.1933T > C	p.Ser654Pro	0	6	
Catalase	fgaCat (Afu2g00200)	c.260 T > A	p.Leu87*	0	6	Protein non-functional
	Cat2 (Afu8g01670)		p.Lys67Thr	0	11	Changes in the function of the protein
	CatB (Afu3g0227)	c.361 A > T	p.Ile121Phe	0	4	Changes in function of protein
Hypoxia	SrbB (Afu4g03460)	c.397 G > A	p.Ala133Thr	0	24	changes in response to hypoxia
Amino-acid metabolism	CpcA (Afu4g12470)	c.439 T > C	p.Ser147Pro	0	4	Amino-acid homeostasis changed
Light sensing	LreB (Afu4g12690)	c.58 C > T	p.Gln20*	0	11	Light-induced morphogenesis changed
		c.1123 C > T	p.Gln375*	0	6	
Hyphal morphology	Gin4 (Afu6g02300)	c.3808 T > C	p.*1270Gln	0	6	Hyphal, conidiation, and virulence in murine model altered
Stress response	Yap1 (Afu6g09930)	c.1249 C > T	p.His417Tyr	0	1	Stress response altered
		c.1004 C > T	p.Thr335Ile	0	1	
Copper transport	CrpA (Afu3g1274)	c.2480 C > G	p.Ala827Gly	0	2	Export of copper improved
		c.585 G > C	p.Gln195His	0	18	Export of copper changed
	Ctr2 (Afu3g08180)	c.539 C > T	p.Ala180Val	0	2	Import of copper improved

Two missense mutations were detected in the cytochrome P450 (CYP450) monooxygenase GliC gene (Afu6g09670) (Table 1), which is involved in the gliotoxin biosynthesis pathway. Interestingly, the nsSNP 1310 A > T resulting in the Asp437Val substitution in GliC was absent in 66 *A. fumigatus* isolates previously described^18^ and is also absent in the eight environmental isolates described here. However, this nsSNP is present in 19 out of 26 dog isolates (Fig. 8). In addition, we found many SNPs in dog isolates causing missense mutations in genes encoding GliT, GliA, and GtmA that are involved in transport (GliA), detoxification of gliotoxin (GliT, GtmA), and downregulation of the expression of the gliotoxin cluster^19–21^.

Various SNPs with HIGH impact were detected in the *pes3* gene *(*Afu5g12730) encoding a multi-modular NRP synthetase of 8515 amino acids^22^ (Table 1). A SNP resulting in a stop codon at amino-acid 1590 (Gly1590*) was found in the isolates belonging to cluster D1 (Fig. 7) and was absent in the environmental isolates (Fig. 8). Similarly, introduction of a stop codon at position 3299 (Trp3299*) was found in isolates of clade C.

Fumitremorgins are tremorgenic mycotoxins of *A. fumigatus* but their role in infection is not clear yet. Interestingly, the encoding FTM cluster is inactive in reference strain Af293 because of an Arg202Leu missense mutation in the FtmD gene (Afu8g00200)^23^. All fungal isolates (i.e., both environmental and dog isolates) did not have this missense mutation, indicating that these isolates might produce fumitremorgin.

### Stress response and reproduction

We investigated which nsSNPs of the dog isolates are present in genes involved in reproduction and stress response (Table 1, Supplementary data set 3). One of the SNPs resulted in a stop codon at Leu87* within the orthologue of the *fgaCat* gene (Afu2g00200). This is a bifunctional catalase-peroxidase involved in the oxidative stress response^17^. Remarkably, this nonsense mutation is present in none of the environmental isolates and only in the 6 high-SNP isolates, which indicate a loss of function of this catalase-peroxidase. Additionally, we found a missense mutation in the mycelial catalase CatB/Cat1 (Afu3g02270) present only in dog isolates from CP2 (DTO 271-A9/B1/B2/B3) (Fig. 8, Table 1). Notably, 24 missense SNPs were present in the light-sensing regulator FphA (Afu6g09260), of which 21 are only present in the high-SNP isolates. This indicates a difference in light sensing between isolates and suggests that light might reach the nasal or frontal sinus^24^.

### TFs with known roles in pathogenesis

A total of 70 missense mutations were found in 17 TFs involved in virulence (Table 1; Supplementary data set 3). However, no disruptive (stop codons) HIGH impact SNPs were present. The Ser147Pro SNP in TF CpcA was only found in dog isolates of subclade A and both isolates of CP9. SNP Ala113Thr on the hypoxia-responsive TF SrbB was present in almost all dog isolates, except for the ones of CP7 and environmental isolates (Figs. 7 and 8). PacC is involved in pH response and is important in the epithelial entry of conidia and invasive growth^10^. This gene had the SNP Arg529Cys and was present in all dog isolates, except for the high-SNP isolates. In addition, we found seven nsSNPs in Yap1 (Supplementary data set 3), of which five only occurred in dog isolates. Two of them (Fig. 8, Table 1) were only present in isolate DTO 271-B9 (Clade D2), suggesting that nsSNPs in this gene might have an impact on oxidative stress response (see below).

### Other SNPs in genes of interest

A high impact SNP was only present in the six high-SNP isolates and results in the loss of the stop codon (*1270Gln) of Gin4 (Afu6g02300). This gene is involved in conidiation, apical compartment length, and virulence in an invertebrate and intranasal murine infection model of IA^25^.

Gene AceA (Afu6g07780) encodes a TF involved in the copper toxicity response and regulates the copper transporters CrpA (Afu3g12740) and Ctr2 (Afu3g08180)^26^. Interestingly, SNPs were found in AceA and CrpA in both environmental and dog isolates (Supplementary data set 3). In the case of CrpA (Fig. 8, Table 1), one SNP was found that was present in 18 dog isolates, and one which was only present in isolates DTO 303-E7 and E9 (clade C). The latter two dog isolates also contained a MODERATE impact nsSNP in Ctr2 (Fig. 8, Table 1). These mutations suggest that in-host adaption to copper stress occurs in dogs.

We did not find alterations in the *cyp51A* gene in the environmental or dog isolates, which is in accordance with our previous observations that none of these dog isolates showed resistance to three tested azoles^4,27^.

### Micro-evolution in the host

NsSNPs previously described in 13 *A. fumigatus* isolates taken from a human patient with CGD over a 2 year-time frame^28^ were compared with the nsSNPs from dog and environmental isolates (Supplementary data set 3). We found an amino-acid change Thr214Ala in the exo-alpha-sialidase Afu4g13800. This protein has been proposed to have a role in the interaction between *A. fumigatus* and its host. This missense SNP is absent in the environmental isolates but present in the six high-SNP variants and in DTO 303-F1 and F2 in clade C and D, respectively (Figs. 7 and 8). In contrast^28^, we did not detect SNPs in Afu6g14720 encoding a putative telomere-associated RecQ helicase and which in human isolates had a broad variety of nucleotide changes (Supplementary data set 3).

### Phenotyping

**Fig. 9:**
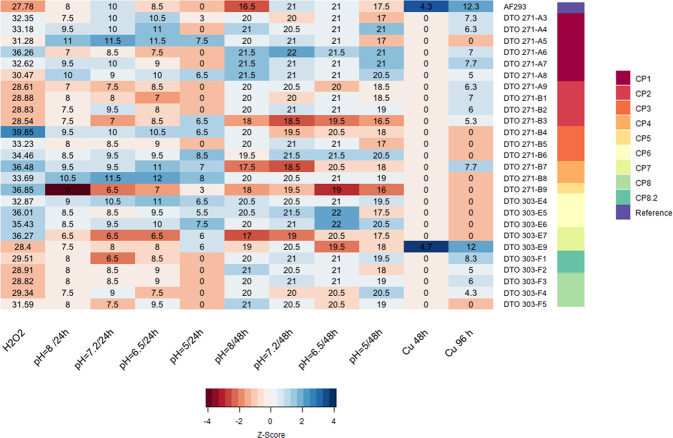
Overview of phenotyping. The color of the cells corresponds to a *z* score of the test (red = poor growth, blue = good growth, white = average growth). Note that in the case of H_2_O_2_ the color relates to the size of the inhibition halo with a red color indicating large inhibition. Average measurements per strain are depicted in each cell. The colored bar to the right indicates the origin of the isolate.

Dog isolates were on average more sensitive to H_2_O_2_ than the reference strain Af293 (Fig. 9). Moreover, differences in H_2_O_2_ sensitivity were observed between isolates from the same fungal plaque. Six isolates (DTO 271-A6 /E5/B4/E7/B7/B9; clades A, B1, C, D2) derived from canine patients (CP1, CP3–CP7, respectively) were very sensitive to H_2_O_2_ (inhibition zones >35 mm) as compared with Af293 (~27 mm) and the other dog isolates (inhibition zones >27 mm and <35 mm) derived from different canine patients (CP1, CP3–CP7, respectively) (Fig. 9). SNPs could not be related to H_2_O_2_ sensitivity. For example, the SNP conferring a stop codon (Leu87*) in the catalase-peroxidase fgaCat (Table 1, Supplementary data set 3) was present in isolates DTO 271-B1/B2/B3/B7/B8/B9 (clade D) but only two of them were considered as highly sensitive (inhibition halo >35 mm) (Fig. 9). A similar situation was observed for the SNPs in the conidial catalases Cat2 (Afu8g01670) and CatB/1(Afu3g0227) (Table 1).

### Fungal growth at different pH values

High variation in growth was observed between dog isolates when cultured at pH 5.0, 6.5, 7.2, and 8.0. Notably, out of 27 dog isolates a total of 21 and 3 isolates showed >20% more growth at pH 8 and pH 5, respectively. In particular, growth at pH 5 for 24 h was very poor for the vast majority of fungal strains. However, after 48 h of growth the difference in colony diameters was considerably reduced when compared with the other pH values (Fig. 9). The Arg529Cys SNP found in PacC could not explain the different growth behavior of isolates. For instance, this SNP was found in several isolates but only DTO 271-B9 did not grow after 24 hours at pH 8 (Fig. 9).

### Copper stress

Isolates from the environment did not grow at all on high copper medium (0.9 mM) during a 2-day period (Supplementary Fig. 2). In contrast, growth at high copper concentration was observed after 4 days in 15 out of 26 dog isolates (Fig. 9 and Supplementary Fig. 2) but not in 12 other isolates of human and environmental origin (Supplementary Fig. 2). These colonies were generally small except for DTO 303-E9 (CP7) that was similar to Af293 (Fig. 9 and Supplementary Fig. 2). Apparently, these two latter strains are the most resistant but 14 other isolates from dogs also showed resistance to high copper. SNPs causing amino-acid substitutions in the copper transporters Ctr2 (Afu3g08180) and CrpA (Afu3g12740) are present in isolate DTO 303-E9, and only in CrpA (Afu3g12740) in DTO 303-F1 (Table 1, Fig. 8) respectively. This suggests a relationship between copper resistance and the presence of the SNPs.

## Discussion

Non-invasive aspergillosis in the SNA of dogs resembles human chronic non-invasive rhinosinusitis that can be caused by several fungal species such as *Aspergillus* species, *Penicillium*, *Mucor*, *Rhizopus*, and phaeohyphomycetes^29^. In dogs *A. fumigatus* is the most commonly identified etiological agent^30,31^. Here, we describe a transcriptomic profiling of natural *A. fumigatus* infections in the sinus of canine hosts using RNA sequencing. In fact, it provides the first transcriptomes resulting from natural infections in animal species with *A. fumigatus*. This study is challenging for several reasons. Differential gene expression cannot be assessed since we are dealing with a variable set of natural infections in dogs of different breeds. In addition, sino-nasal fungal plaques represent large three-dimensional structures that differ in location and they are most commonly found in the nasal cavity or frontal sinus. The time of infection and their micro-environments also differ. Knowledge of SNA is limited, and most studies have focused on diagnostics and treatment of dogs^5,30,32–34^, on fungal resistance^27^ and on the local immune response^6–8^. These studies suggested an induction of Th1 response during SNA. In addition, interleukin IL-6 was upregulated in dogs with SNA^8^. IL-6 is related to the Th17 response that promotes antifungal properties of neutrophils^35^ and is required for optimal fungal clearance at the mucosal level^36,37^. Microarray analysis showed no differential expression of genes involved in the Th17 response^6^. This may be explained by the fact that these studies used different dog breeds. The fact that these studies did not analyze individual patients but made use of pools of RNA of different patients makes it difficult to find the cause of the difference in the qPCR and microarray studies. In contrast, we did analyze individual patients enabling analysis of individual immune responses. Of the nine fungal plaques isolated over a period of 2 years, we only obtained RNA from four samples that passed the rigorous quality check required for RNAseq. The data obtained from the same dog showed high similarity in gene expression. Nevertheless, comparison of transcriptomes from more dogs are required to address genetic heterogeneity among dogs further. Our analysis are in agreement with previous qPCR studies^7,8^ and we found upregulation of factors involved in the Th17 response (i.e., STAT3 and the Il-23 subunit P19). Yet, expression of IL-17 was detected in only one, a Saint Bernard breed (CP7). Thus, the antifungal Th17 response seems to be affected in SNA in certain breeds. A defective Th17 response can result in reduced recruitment of neutrophils to the site of infection. However, strong or prolonged activation of the Th17 response can also cause the opposite effect resulting in an excessive inflammation and immune pathology^36,38^ Kynurenines may be involved in the dysregulation of the Th17 response at the mucosal surface and in the establishment of the chronic status of SNA in dogs. These molecules result from the degradation of tryptophan via indoleamine 2,3-dioxygenase (IDO1) and play a role in control and establishment of fungal infections^39^. Both host and *A. fumigatus* have IDO1 genes and can thus produce kynurenine. It has been reported that kynurenines produced by *A. fumigatus* can inhibit IL-17 production in human monocyte-derived macrophages^40^. Furthermore, the formation of host-derived kynurenine on paracoccidioidomycosis was shown to modulate the immune response such that a state of disease tolerance was created, thereby preserving host fitness without pathogen clearance^39,41^.

The continuous proinflammatory reaction in the dog epithelium can be stimulated by the release of S100 proteins. These proteins have pleiotropic effects on nutritional immunity and induction of proinflammatory responses^42^. Moreover, expression of pattern recognition receptors involved in the innate immune response to *A. fumigatus* like TLR (toll-like receptor) 2 and 4^43^, Mincle (CLEC4E)^44^, and Dectin-1 (CLEC7A)^45^ accompanied by high expression of IL-8, which can be stimulated by IL-1α, hyphal antigens^46,47^, and Dectin-1^47^, contributes to the continuous activation of a proinflammatory reaction and a Th1 response. The fact that IL-10 was shown to be expressed in SNA may result in a dampened Th1 response that would prevent excessive tissue damage. Moreover, it can contribute to the chronic state of the infection as suggested previously^7^. Furthermore, adaptive responses of *A. fumigatus* enable growth and contribute to the suppression of the host immune response. This includes generation of immune-modulatory metabolites, adaptation to nutritional stress, and phenotypic and genetic plasticity. For instance, full expression of the gliotoxin, neosartoricin and hexadehydroastechrome clusters was found. Gliotoxin has several immune-suppressive activities, killing of immune cells and inhibition of H_2_O_2_ production in macrophages^48^. Furthermore, gliotoxin together with neosartoricin can suppress the local adaptive immune response via inhibition of T-cell proliferation^49^. nsSNPs were found in fungal genes related with detoxification of reactive oxygen species produced by the host, and in genes involved in the pH and stress response. These host environmental factors can play a role in the selection of variants during in-host adaptation. For example, nsSNPs were found in the copper exporter CrpA and the copper importer Ctr2 in some strains that were able to grow on high concentrations of copper. In addition, expression data from the fungal plaques showed that CrpA had a highly variable expression between all four fungal plaques, which also carried isolates with the nsSNP in the mentioned gene. This suggests that high copper concentrations at the mucosal surface impose a selection of copper-resistant variants. It is not clear how high copper concentration may develop but the metal may be released from macrophages that are known to accumulate copper. Copper is a transition metal that is used by the macrophages to produce hydroxyl radicals in a Fenton reaction to kill intracellular pathogens^26,50^. Cytotoxic activity of secondary metabolites like gliotoxin may be responsible for release of the copper into the environment thus increasing copper concentration at the mucosal surface.

Zinc is another metal that has an important role in fungal infections, for example, host calprotectin is a heterodimer of S100A8/A9 proteins, which can sequestrate zinc, making it unavailable for the fungus^51^. Therefore, fungi have developed mechanisms for the uptake and homeostasis of this metal. In the case of *A. fumigatus* ZrfA, ZrfB, and ZrfC are zinc transporters, and AspF2 (a protein orthologue to the *C. albicans* zincophore PRA1)^52,53^. The interaction between ZrfC and AspF2 is of our interest because the former regulates the transport of zinc under alkaline conditions^52^, and the latter is hypothesized (in *C. albicans*) to bind to available zinc in the host–pathogen interphase and transfer it to Zrt1^53,54^. In our RNAseq data, ZrfC and AspF2 were expressed in a high stable and medium stable fashion. This observation complements the fact that all fungal plaques presented a pH of 8 as determined by pH measurement directly after isolation of the plaques from the patient. We propose that alkalization of the sino-nasal area is part of the fungal strategy to colonize and counter the host nutritional immunity. Another remarkable phenotype of the fungal biofilm is the absence of asexual reproduction, the fungal plaques appear as white structures and microscopic analysis only scarcely showed some formation of conidiophores^4^ interestingly, expression analysis indicated no expression of the *abaA* gene encoding a TF that together with TFs BrlA and WetA are required for asexual reproduction^55^ Both *brlA* and *wetA* are medium variable expressed in the four biofilms (Supplemental material S2). These results explain why asexual reproduction is not observed. Why *abaA* is not expressed remains to be determined but might be linked to environmental factors in the sinus that influence asexual reproduction.

Together, our results indicate that the interaction between the developing *A. fumigatus* biofilm and the mucosal immune system ends up in the formation of a sino-nasal warzone. The existence of genetic variants within a fungal biofilm is expected to be the result of selection in this stressful environment of the dog’s nasal cavity and frontal sinus. Generation of individual cells with an increased frequency of mutation can be advantageous for the pathogen in a stressful environment. As such, the situation in SNA would be similar to the one encountered in chronic infections like cystic fibrosis in which hypermutable variants of *Pseudomonas aeruginosa* were described^56–58^. This phenomenon has previously also been described for the fungal species *Candida albicans*^59^ and *Cryptococcus neoformans*^60,61^ and this would be the first report for *A. fumigatus*. Increased mutation rate in our study may be owing to a defective DNA repair system as indicated by the finding that the genes involved in this system show increased incidence of biallelic non-synonymous SNPs. In addition, formation of heterokaryons in the fungal plaque may increase the presence of alternative alleles^62^.

## Methods

### Strains

A total of 34 *A. fumigatus* isolates were used in this study (Table 2), 8 indoor environmental isolates from the Netherlands, 26 isolates from dog patients with SNA. The 26 isolates were obtained from fungal plaques derived from 8 canine patients (CP1-8) suffering from SNA^4^ (Supplementary Table 1). Part of these plaques were immediately frozen in liquid nitrogen and stored at −80°C for RNA isolation and sequencing. Isolates from dogs were obtained with owner’s consent applying a standardized protocol at Veterinary Medicine, Utrecht University, The Netherlands.

**Table 2:** Overview of *A. fumigatus* isolates.

	No. of isolates	Provided by	Origin	Reference/year
Environmental	8	Westerdijk Insitute Utrecht	The Netherlands, Indoor air	Valdes et al. (2018)^4^ 2005–2008
Dog	26	Clinical Sciences of Companion Animals, Veterinary Medicine, Utrecht University	The Netherlands	Valdes et al. (2018)^4^ 2010–2012

### RNA isolation and sequencing

Material of fungal plaques (100–200 mg) was homogenized for 5 min with two metal balls (4.76 mm in diameter) in a Tissuelyzer at 20 Hz min-1 (Qiagen, Venlo, The Netherlands) in the presence of 1 mL TRIzol (Thermo Fisher Scientific, USA). After homogenization, the mixture was incubated for 5 min at RT, followed by the addition of 200 µL chloroform and subsequent incubation at RT for 3 min. Samples were centrifuged for 10 min at 4 °C at 10,000 × *g* and the RNA in the aqueous phase was isolated and purified using the RNeasy RNA kit (Qiagen).

RNA sequencing was performed at GenomeScan (Leiden, The Netherlands) using Illumina NextSeq 500 according to manufacturer protocols. In brief, RNA quality was checked using the Fragment Analyzer. Libraries were made using the MEBNext Ultra Directional RNA Library PrepKit for Illumina. The size of the cDNA fragments was confirmed to be in the 300–500 bp range and 1.6 pM of DNA was used for sequencing. RNA sequence data are available in the NCBI Sequence Read Archive (SRA) under the BioProject ID PRJNA557110

### RNA sequencing analysis

Quality of raw reads was checked using FastQC (https://www.bioinformatics.babraham.ac.uk/projects/fastqc/), whereas cleaning and trimming were performed using Fastx-Toolkit (http://hannonlab.cshl.edu/fastx_toolkit/). CanFam3.1 transcripts from Ensembl (https://www.ensembl.org/Canis_familiaris/) and *A. fumigatus* Af293 transcripts from AspGD (http://www.aspergillusgenome.org/) served as reference to quantify expression using Kallisto^63^. Transcripts per million (TPM) was used as unit of expression. Transcripts with a TPM > 1 in all four samples were considered expressed.

### Functional categorization and enrichment of expressed transcripts

Gene ontology (GO) and KEGG enrichment analysis was performed using the R package gProfileR^64^, with best term per parent group (strong filtering). REVIGO (http://revigo.irb.hr/) was used for removing redundancy in GO terms. FunCat ontology was determined using FungiFun2^65^. Additionally, published lists of *A. fumigatus* genes involved in different processes were used to query the transcriptomes (Table 3).

**Table 3:** Sets of *Aspergillus fumigatus* genes involved in biological processes.

Category	Reference
Transcription factors involved in virulence and pathogenesis	Bultman et al. 2017^16^
Reproduction and stress response	de Vries et al. 2017^17^
Aspergilli stress response	Miskei et al. 2009^14^
Secondary metabolism	Khaldi et al. 2010^13^
Genes involved in-host–pathogen interaction	Urban et al. 2017^15^

### Comparison with reported sino-nasal aspergillosis microarray data

Expression data^6^ were extracted from the array express database using the R package Array express V1.42.0. Raw data were processed and reanalyzed using the RMA method contained in the R package affy V1.60.0. Expression values of each transcript corresponded to the mean value of expression of the probes targeting that particular transcript. Transcripts were considered differentially expressed when they showed a Log2 Fold change (control vs. affected dogs) > ±2 (up, downregulation, respectively).

### Fungal culturing for genomic DNA isolation

Genomic DNA was isolated using a modification of the protocol of Lee et al.^66^. In brief, 200 µl of conidial suspensions^67^ were seeded in potato dextrose agar (PDA) for 7 days at 37 °C. Conidia from these cultures were inoculated into 20 mL of potato dextrose broth, followed by incubation for 3–4 days at 37 °C in an Erlenmeyer. Mycelia were harvested by filtration over three layers of sterile Miracloth (Merck, Darmstadt, Germany), washed three to four times with sterile H_2_O, and dried using sterile paper towel at room temperature. Samples were lyophilized, followed by pulverization using sterile mortar and pestle. Ground samples (~30 mg) were used for DNA isolation using Qiagen DNeasy PowerPlant Pro Kit following manufacturer recommendations for problematic samples. DNA concentration and quality were checked using the Qubit® assay system

### Whole-genome sequencing of fungal isolates

Whole-genome sequencing was performed by the Utrecht sequencing facility. In brief, libraries were prepared using Truseq DNA Nano library and sequenced on an Illumina NextSeq 500 with150 bp pair end mid output configuration. DNA sequence data are available in the NCBI SRA under the BioProject ID PRJNA598656

### Sequence analysis

Quality of raw reads was checked using fastQC (https://www.bioinformatics.babraham.ac.uk/projects/fastqc/). Cleaning and trimming were performed using Fastx-Toolkit (http://hannonlab.cshl.edu/fastx_toolkit/). Reads were mapped to the genome of reference strain Af293 (Genome version from AspGD: s03-m05-r07 http://www.aspergillusgenome.org/) with bowtie2 v2.2.9 using options end to end and very sensitive. SAMtools v1.3 was used for further quality control. Freebayes v0.9.10-3^68^ with option ploidy 1 was used for variant calling. Post filtering of the vcf file was performed using vcfilter (“qual >20”, depth 5×).

### Identification of variants related to *A. fumigatus* evolution in the host

Mutation analysis in dog isolates was performed using association tests, core/non-core SNP analysis, and variant selection and annotation per group of interest (Supplementary Fig. 3).

#### Variant annotation

The filtered vcf file was used as input for SnpEff v4.3k in order to predict the effect of the variants^69^. For downstream analysis HIGH (gain or loss of stop codon, splice region variant) or MODERATE (missense) effects were used.

#### Core/non-core variants

A variant that was found in all isolates of a group was considered a core variant, while variants that were different in at least one isolate were classified as non-core variant. FungiFun2^65^ was used to perform FunCat enrichment of core/non-core variants having a HIGH impact (for example loss of start or stop codon).

### A targeted search of variants affecting genes of interest

SNPs present in genes of interest^13–17^ were compared with whole-genome sequencing data of 66 isolates of *A. fumigatus*^18^. To this end, the full annotated vcf file (environmental and dog isolates) was used and SNPs with a HIGH and MODERATE effect were taken into account.

### Phylogenetic tree

A phylogenetic tree was constructed based on the filtered biallelic SNPs with no missing data using the R package SNPRelate v3.7^70^. The SNPs were pruned using thresholds reported for *A. fumigatus* (linkage disequilibrium threshold of 0.8 and a minor allele frequency of 0.03)^18^. MEGA X^71^ was used for the calculation of the phylogenetic tree. In brief, a multi-fasta file with the SNP per strain was aligned with MUSCLE^72^ and the resulting alignment was used for statistical test of best-fit model of nucleotide substitution. The generalized time-reversible (GTR) model resulted in the best fit using the corrected Akaike information criterion. A total of 3826 pruned marker SNPs was used to build a maximum likelihood phylogenetic tree based on the GTR substitution model with 1000 bootstrap replicates.

### Phenotyping of *A. fumigatus* isolates from SNA fungal plaques

Growth of the dog isolates was assessed during oxidative (H_2_O_2_), pH and copper stress. Prior to the phenotypical tests, strains were grown at 37 °C on PDA. Spores were harvested with Milli-Q® (Millipore Corp) water and filtered through three layers of sterile Miracloth (Merck, Darmstadt, Germany). They were diluted to 1 × 10^8^ ml^−1^ and stored at 4 °C for maximally 2 weeks.

#### pH growth test

Minimal agar medium^73^ with 25 mM glucose (GMMA) was titrated at pH 5.0, 6.5, 7.2, and 8.0^10^. Wells of 12-well plates (Greiner Bio One International GmbH) were filled with 2 ml medium, inoculated with 10^3^ conidia in the center of each well, and incubated at 37 °C. Colony diameter was measured after 24 and 48 hours using biological duplicates.

#### Copper stress

Copper excess agar (GMMA-Cu containing 60 mM glucose and 0.9 mM Cu^2+^) was prepared by dissolving 0.015 g anhydrous copper sulfate (Merck, Darmstadt, Germany) in 47 ml MM, followed by addition of 3 ml 1 M glucose. GMMA-Cu (2 ml) was added to wells of 12-well plates, inoculated with 10^8^ conidia in the center of the wells and incubated at 37 °C up to 4 days. GMMA (containing 1.25 µM Cu^2+^) was used as negative control. Colony size was determined in biological triplicates.

#### Hydrogen peroxide stress

A two-layered agar plate (60 mm Petri dish) was used containing 100 µl 500 mM H_2_O_2_ in a 5 mm wide hole in the center. The bottom layer consisted of 10 ml GMMA and a top layer of 5 ml GMMA containing five 10^7^ conidia (conidia were always 2 weeks old). After 16 hours of incubation, diameter of inhibition zones (as biological triplicates) was determined using ImageJ 1.52a^74^.

### Reporting summary

Further information on research design is available in the Nature Research Reporting Summary linked to this article.

## Supplementary information

Reporting Summary Checklist

Supplementary Information

Supplementary Data Set 1

Supplementary Data Set 2

Supplementary Data Set 3

## Data Availability

DNA sequence data are available in the NCBI SRA under the BioProject ID PRJNA598656. RNA sequence data are available in the NCBI SRA under the BioProject ID PRJNA557110. All other relevant data are available from the corresponding author on request.
