# Supplementary material for: The sino-nasal warzone: transcriptomic and genomic studies on sino-nasal aspergillosis in dogs
Source: NPJ Biofilms Microbiomes. 2020 Nov 12;6:51. doi: 10.1038/s41522-020-00163-7 (PMC7665010; doi:10.1038/s41522-020-00163-7)
Supplement: Supplementary file 1 — Reporting Summary Checklist [file 41522_2020_163_MOESM1_ESM.pdf]

nature research

Corresponding author(s): NPJBIOFILMS-00802

Last updated by author(s): 2020-9-9

Reporting Summary

Nature Research wishes to improve the reproducibility of the work that we publish. This form provides structure for consistency and transparency in reporting. For further information on Nature Research policies, see our Editorial Policies and the Editorial Policy Checklist.

Statistics

For all statistical analyses, confirm that the following items are present in the figure legend, table legend, main text, or Methods section.

n/a	Confirmed	
☐	☒	The exact sample size (*n*) for each experimental group/condition, given as a discrete number and unit of measurement
☐	☒	A statement on whether measurements were taken from distinct samples or whether the same sample was measured repeatedly
☒	☐	The statistical test(s) used AND whether they are one- or two-sided<br>*Only common tests should be described solely by name; describe more complex techniques in the Methods section.*
☒	☐	A description of all covariates tested
☒	☐	A description of any assumptions or corrections, such as tests of normality and adjustment for multiple comparisons
☐	☒	A full description of the statistical parameters including central tendency (e.g. means) or other basic estimates (e.g. regression coefficient) AND variation (e.g. standard deviation) or associated estimates of uncertainty (e.g. confidence intervals)
☒	☐	For null hypothesis testing, the test statistic (e.g. *F*, *t*, *r*) with confidence intervals, effect sizes, degrees of freedom and *P* value noted<br>*Give P values as exact values whenever suitable.*
☒	☐	For Bayesian analysis, information on the choice of priors and Markov chain Monte Carlo settings
☒	☐	For hierarchical and complex designs, identification of the appropriate level for tests and full reporting of outcomes
☒	☐	Estimates of effect sizes (e.g. Cohen's *d*, Pearson's *r*), indicating how they were calculated

Our web collection on statistics for biologists contains articles on many of the points above.

Software and code

Policy information about availability of computer code

Data collection	Ensembl,AspGD(http://www.aspergillusgenome.org/) ,R package Array express V1.42.0.
Data analysis	FastQC (https://www.bioinformatics.babraham.ac.uk/projects/fastqc/),Fastx-Toolkit (http://hannonlab.cshl.edu/fastx_toolkit/),Kallisto,R package affy V1.60.0,R package gProfileR,REVIGO (http://revigo.irb.hr/),FungiFun2(https://elbe.hki-jena.de/fungifun/),Bowtie2 v2.2.9,SAMtools v1.3,Freebayes v0.9.10-3,SnpEff v4.3k,R package SNPRelate v3.7,MEGA X,ImageJ 1.52a

For manuscripts utilizing custom algorithms or software that are central to the research but not yet described in published literature, software must be made available to editors and reviewers. We strongly encourage code deposition in a community repository (e.g. GitHub). See the Nature Research guidelines for submitting code & software for further information.

Data

Policy information about availability of data

All manuscripts must include a data availability statement. This statement should provide the following information, where applicable:

- Accession codes, unique identifiers, or web links for publicly available datasets
- A list of figures that have associated raw data
- A description of any restrictions on data availability

DNA sequence data is available in the NCBI Sequence Read Archive (SRA) under the BioProject ID PRJNA598656. RNA sequence data is available in the NCBI Sequence Read Archive (SRA) under the BioProject ID PRJNA557110. All other relevant data are available from the corresponding author on request.

Field-specific reporting

Please select the one below that is the best fit for your research. If you are not sure, read the appropriate sections before making your selection.

☒ Life sciences ☐ Behavioural & social sciences ☐ Ecological, evolutionary & environmental sciences

For a reference copy of the document with all sections, see nature.com/documents/nr-reporting-summary-flat.pdf

Life sciences study design

All studies must disclose on these points even when the disclosure is negative.

Sample size	we obtained from eight canine patients (one dog was re-infected) with SNA 26 fungal isolates which were used in genetic characterization. from 4 patients we could isolated sufficient RNA of good quality
Data exclusions	no data were excluded
Replication	sequencing of genomes was covered 23.6. Sequencing of RNA from four fungal plaques was performed once for each sample which were each unique samples
Randomization	not relevant for this study
Blinding	blinding was not relevant, the study encompasses expression studies, DNA sequencing and pehnotypic characterization of isolates

Reporting for specific materials, systems and methods

We require information from authors about some types of materials, experimental systems and methods used in many studies. Here, indicate whether each material, system or method listed is relevant to your study. If you are not sure if a list item applies to your research, read the appropriate section before selecting a response.

Materials & experimental systems

n/a	Involved in the study	
☒	☐	Antibodies
☒	☐	Eukaryotic cell lines
☒	☐	Palaeontology and archaeology
☐	☒	Animals and other organisms
☒	☐	Human research participants
☒	☐	Clinical data
☒	☐	Dual use research of concern

Methods

n/a	Involved in the study	
☒	☐	ChIP-seq
☒	☐	Flow cytometry
☒	☐	MRI-based neuroimaging

Animals and other organisms

Policy information about studies involving animals; ARRIVE guidelines recommended for reporting animal research

Laboratory animals	no laboratory animals were used
Wild animals	no wild animals were used
Field-collected samples	samples were collected from canine patients presented at the veterinary hospital at Utrecht University
Ethics oversight	informed consent via standard protocols was obtained from the owners of the dogs

Note that full information on the approval of the study protocol must also be provided in the manuscript.
