# Supplementary material for: The sino-nasal warzone: transcriptomic and genomic studies on sino-nasal aspergillosis in dogs
Source: NPJ Biofilms Microbiomes. 2020 Nov 12;6:51. doi: 10.1038/s41522-020-00163-7 (PMC7665010; doi:10.1038/s41522-020-00163-7)
Supplement: Supplementary file 2 — Supplementary Information [file 41522_2020_163_MOESM2_ESM.pdf]

Supplementary figures.

Supplementary data set 1. Excel file with RNA seq statistics (number of reads and % aligned) from mapping RNA-seq reads to *C. lupus familiaris*, GO enrichment analysis mapping to innateDB and comparison to a previous microarray dataset is also included. (.xlsx file)

Supplementary data set 2. Excel file with RNA seq statistics (number of reads and % aligned) from mapping RNA-seq reads to *A. fumigatus*, GO enrichment analysis and mapping to datasets in table 3.(.xlsx file)

Supplementary data set 3. Excel file with WGS coverage, SNP’s analysis, association test tables, and locations of particular SNP’s on gene of interest. (.xlsx file)

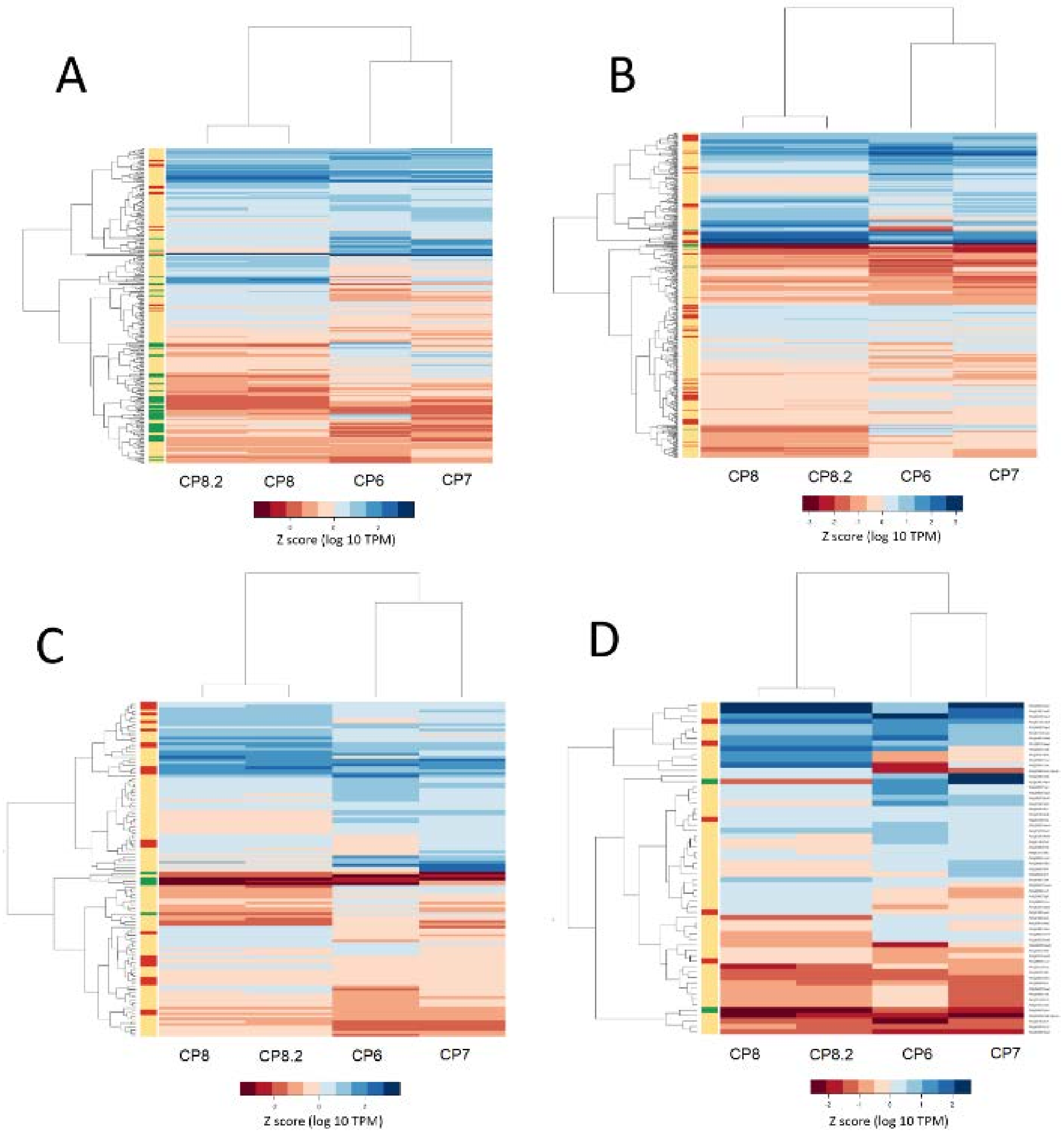

A
CP8.2
CP8
CP6
CP7
Z score (log 10 TPM)
B
CP8
CP8.2
CP6
CP7
Z score (log 10 TPM)
C
CP8
CP8.2
CP6
CP7
Z score (log 10 TPM)
D
CP8
CP8.2
CP6
CP7
Z score (log 10 TPM)

Supplementary figure 1. Heatmaps with clustering of total expression of genes implied in secondary metabolism (A), stress response (B), pathogen host interaction (C) and transcription factors related with virulence and reproduction and stress (D). The color code under the graphs represent Z scores of TPM. The color bar along the side of graphs A-D indicates the variability based on the CV representing low (red), medium (yellow) or highly variable (green) genes.

Supplementary Table 1. Additional information of the Canine patients.

Dog	Breed	sex	Age (when sampled)
CP6	Labrador Retriever	Male (entire)	3
CP7	Saint Bernard	Male (neutered)	6
CP8 and 8.2	Golden Retriever	Female (neutered)	5 (CP8) and 7 (CP8.2)

All dogs lived in or near a rural area. Patients were presented at the clinic by the owners when clinical signs became apparent. Fungal plaques were obtained via trephination of the frontal sinus and/or rhinoscopic removal in the nasal sinus with a 30 degrees 2.7 mm rigid endoscope and using a combination of suction and a metal hook. Trephination was performed if mycotic plaques in the frontal sinus were diagnosed by CT. After removal of the fungal plaques the nasal sinus was flushed in most cases with clotrimazole for 15 min each in ventral, left and right lateral, and dorsal recumbency, followed by flushing for 15 min in ventral recumbency, after which the clotrimazole was allowed to leave the nasal sinus by gravity (head down). More information in reference 4 (Valdes, I. D. *et al*. Comparative genotyping and phenotyping of *Aspergillus fumigatus* isolates from humans, dogs and the environment. *BMC Microbiol.* **18**, 118-2 (2018)).

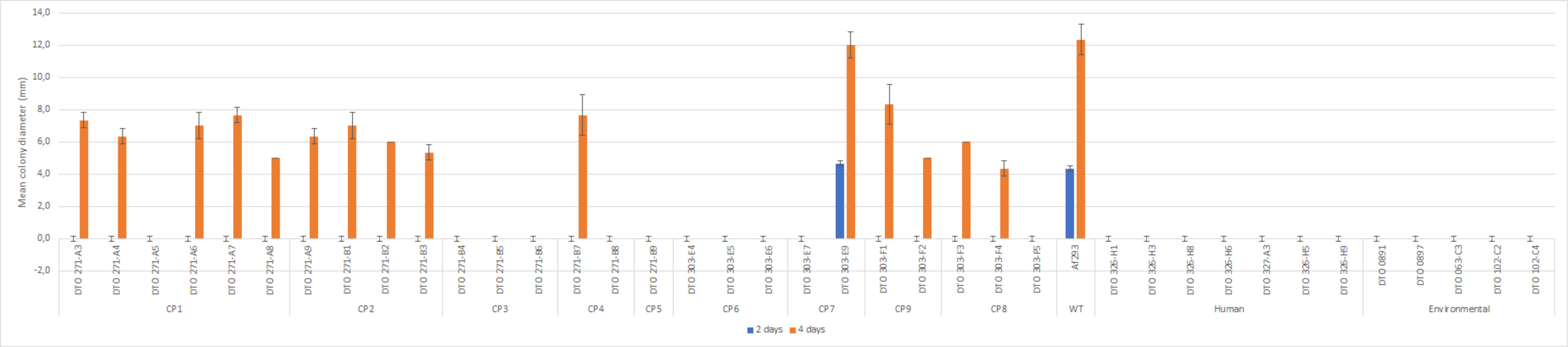

Supplementary figure 2. Mean colony diameter (mm) and standard error of 26 dog isolates compared to Af293 as well as a set of *A. fumigatus* isolates from humans or environment. Plates were incubated at 37°C for 2 and 4 days with excess copper (0.9 mM) as described in Materials and Methods. All fungal isolates (dogs, human and environment) are described in detail in ref 4 (Valdes, I. D. *et al*. Comparative genotyping and phenotyping of *Aspergillus fumigatus* isolates from humans, dogs and the environment. *BMC Microbiol.* **18**, 118-2 (2018)).

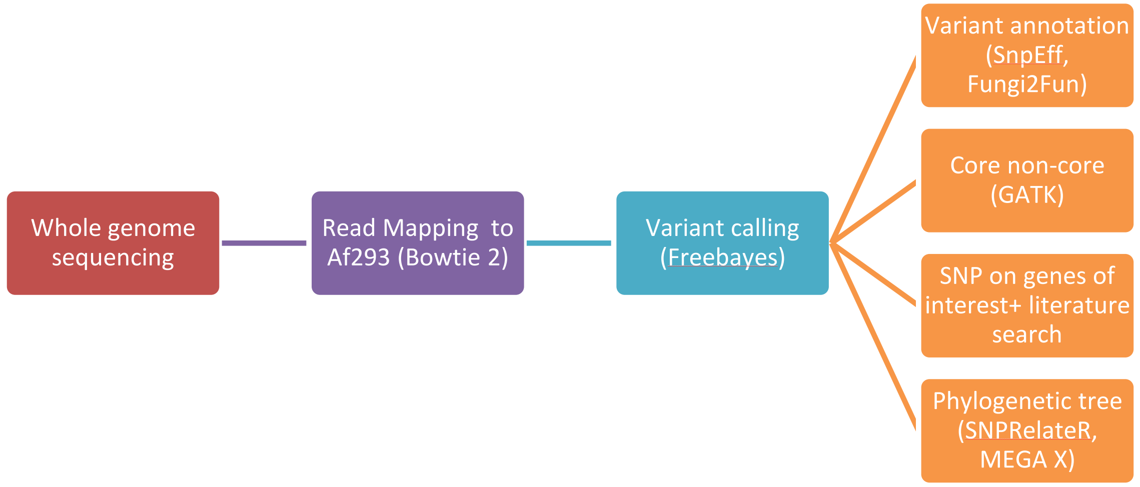

Supplementary figure 3. Scheme of the workflow of sequence analysis of *A. fumigatus* dog isolates
